# RETRACTED: Kaya et al. The Superiority of Compressed Colchicine Tablets over Coated Colchicine Tablets for Familial Mediterranean Fever. *Medicina* 2024, *60*, 1728

**DOI:** 10.3390/medicina61112052

**Published:** 2025-11-18

**Authors:** Mehmet Nur Kaya, Muhammed Canbaş, Özlem Kılıç, Abdullah Doğan, Sedat Yılmaz

**Affiliations:** Rheumatology Department, Gülhane Training and Research Hospital, University of Health Sciences Turkey, 38000 Ankara, Turkey

The Journal retracts the article “The Superiority of Compressed Colchicine Tablets Over Coated Colchicine Tablets for Familial Mediterranean Fever” [1] cited above.

Following publication, concerns were brought to the attention of the Editorial Office regarding an overlap with a previously published manuscript [2] with a different authorship group.

Adhering to our complaint procedure, an investigation was conducted by the Editorial Office and Editorial Board that confirmed the presence of significant overlaps in the Discussion section, Tables 3 and 4, and the references’ accession dates between the above-mentioned manuscripts. As a result, the authors, the Editorial Office, and the Editorial Board have decided to retract this article [1] as per MDPI’s retraction policy (https://www.mdpi.com/ethics#_bookmark30).

This retraction was approved by the Editor-in-Chief of the journal *Medicina.*

The authors agreed to this retraction.
